# Glycemic Variability and Control by CGM in Healthy Older and Young Adults and Their Relationship With Diet

**DOI:** 10.1210/jendso/bvaf081

**Published:** 2025-05-08

**Authors:** Anika Köhlmoos, Manuela Dittmar

**Affiliations:** Human Biology, Zoological Institute, Christian-Albrechts-University, Kiel 24118, Germany; Human Biology, Zoological Institute, Christian-Albrechts-University, Kiel 24118, Germany

**Keywords:** healthy aging, continuous glucose monitoring (CGM), glycemic variability, glycemic control, nutrition

## Abstract

Continuous glucose monitoring (CGM) might be beneficial for investigating healthy aging since high glycemic variability may increase protein glycation, oxidative stress, and inflammation, resulting in vascular damage. Additionally, CGM data on the risks for hypoglycemia and hyperglycemia are scarce, have not been analyzed by individual day and night blocks, and have not been related to diet. Therefore, this study aimed to compare glucose parameters of healthy older and young adults and the relationship with diet. Participants were 34 young (age 20-35 years) and 27 older volunteers (age 60-75 years) with a normal glycated hemoglobin A_1c_ less than 39 mmol/mol hemoglobin, free of disorders and medication. Twenty-four CGM-derived glucose parameters measured over 5 consecutive days were analyzed for whole days and for individual daytime and nighttime blocks. Dietary intake was determined by 3-day dietary record. Neither intraday nor interday glycemic variability differed between the healthy age groups. Glycemic control was good in both age groups, but somewhat poorer in older adults. The risk of hyperglycemia was higher and of hypoglycemia lower in older adults. During the daytime, mean and minimum glucose were higher in older adults. During the nighttime, age group differences were small. The carbohydrate intake correlated positively with glycemic variability in both age groups. The protein intake correlated positively with the hypoglycemic risk in young adults, but negatively in older adults. Results suggest that healthy aging does not increase glycemic variability and the risk of hypoglycemia. The effect of diet on hypoglycemic and hyperglycemic risk might change with aging.

Glycemic variability describes the fluctuation in blood glucose concentration throughout the day [1]. Glycemic control provides information on how well a person maintains the glucose concentration within the desired euglycemic range, avoiding both hypoglycemic and hyperglycemic episodes [2]. Therefore, the investigation of glycemic variability and glycemic control informs about the glycemic state of a person. The blood glucose concentration correlates highly with the glucose concentration in the interstitial fluid of the subcutaneous tissue [1]. The tissue glucose concentration can be measured in real time over a period of several days by continuous glucose monitoring (CGM) through a glucose sensor [3]. CGM-derived measurements inform about glycemic variability and glycemic control, as defined earlier. They are used for optimizing therapy in patients with diabetes [4]. However, CGM may be beneficial for healthy people [5], because a normal fasting glucose value and a normal long-term glucose value (glycated hemoglobin A_1c_ [HbA_1c_]) alone does not inform about glycemic variability and about the number of hyperglycemic and hypoglycemic episodes that may be harmful to the body. Especially for healthy older people, knowledge on the number of hypoglycemic and hyperglycemic episodes is important. A high glycemic variability with many hyperglycemic and hypoglycemic episodes may increase protein glycation, oxidative stress, and inflammation, resulting in vascular damage [6-8]. Hypoglycemia may lead to memory problems and fainting, and hyperglycemia may damage in the long-term kidneys, the cardiovascular system, the nervous system, and the eyes [9, 10]. Knowledge on glycemic variability in healthy older people would therefore allow reducing their risk for developing these diseases.

CGM studies in healthy adults were conducted predominantly in young groups (age 20-39 years), middle-aged groups (age 40-59 years), and mixed-age groups including older individuals (eg, [11-13]). In contrast, CGM data from apparently healthy groups older than 60 years are scarce [14-17]. Those studies performed CGM only over a 24-hour period [14] or did not report time of glucose concentration in the hypoglycemic, hyperglycemic, or target range [14-16] or did not assess glucose parameters separately for day and night blocks [17]. Knowledge on day-to-night fluctuations in the glucose concentration is particularly relevant for older people, since glucose variation and its regulation weaken with aging [18, 19].

Therefore, the first objective of the present study was to provide data on glycemic variability and glycemic control by CGM, averaged over 5 consecutive whole days and day-night blocks in healthy older adults with normal HbA_1c_ and to compare the glucose values with those of healthy young adults to provide information on healthy aging.

Several parameters such as diet may influence glucose concentration and variability [20]. The timing and composition of diet (amounts of carbohydrates, proteins and fats, and their ratios) influence the glucose fluctuation in blood and tissue [20]. Santos-Báez and colleagues [21] reported in a small group of mixed ages a positive correlation between carbohydrate intake and different parameters for glycemic variability and a negative correlation with protein intake. However, no study has examined the influence of dietary composition on glycemic variability and glycemic control by CGM in groups of healthy older people older than 60 years. Therefore, the second objective of this study was to analyze the relationship of dietary composition with glycemic variability and glycemic control by CGM in healthy older and young adults.

## Materials and Methods

### Participants

Participants were 27 older (63% women; age 60-75 years) and 34 young (47% women; age 20-35 years) healthy volunteers of European ancestry. They were recruited through flyers and public advertisements. All participants had to be healthy. The inclusion and exclusion criteria were assessed by medical history, questionnaire, and laboratory analysis (HbA_1c_). Exclusion criteria were any acute and chronic illness, injury or inflammation, cancer, depression or psychiatric diseases, sleeping orders, apnea, or use of medication and exogenous melatonin. The volunteers had an HbA_1c_ value less than 39 mmol/mol Hb (<5.7%), were nonsmokers, and presented with a body mass index (BMI) between 18.5 and 29.9 kg/m^2^. They had a regular sleep-wake pattern and a usual sleep duration of 6 to 9 hours, did not shift work, and had no jet lag. They had at least 3 meals a day, did not present with eating disorders, and did not follow a weight-reducing diet. Women who were pregnant or lactating as well as competitive athletes were not included in the study. All volunteers signed informed consent forms. The study protocol (D 533/23) was approved by the human ethics committee at the Medical Faculty of the Christian-Albrechts-University, Kiel. The study was performed in accordance with the Declaration of Helsinki.

### Procedures

Data collection took place from July 2023 to January 2024 in northern Germany. Volunteers who met the inclusion criteria visited the Department of Human Biology, where they got information about the study procedure. Then HbA_1c_ was measured, followed by anthropometry and estimating body composition. The individual study period consisted of 6 days during which each participant wore a CGM sensor on the upper arm. CGM data from day 1 were discarded since the accuracy of the CGM system is lower the first day [3]. CGM data from days 2 to 6 were included in the analyses. The participants noted on a data sheet the times of turning the lights off in the evening and on in the morning. They were further informed on how to fill out a dietary record on days 4 to 6. They were instructed to maintain their usual dietary habits and physical activity level, but should refrain from drinking alcohol, which affects sleep quality [22]. After the 6 study days, the participants returned all devices to the university. The glucose sensor was removed and data were read from the glucose receiver for further analysis.

### Assessment of Anthropometrical and Body Composition Characteristics

Body weight, height, and abdomen circumference were determined with an electronic scale, a wall-mounted measuring device, and a measuring tape, respectively. BMI was calculated as weight (kg)/height^2^ (m^2^). Body composition was analyzed using a tetrapolar bioelectrical impedance analyzer (Nutriguard-MS, Data-Input GmbH) and the software Nutri Plus version 5.4.1 by the manufacturer.

### Continuous Glucose Monitoring

CGM was performed for 6 days using the G7 glucose sensor (Dexcom Inc). The sensor was inserted on the nondominant upper arm of the participants. It measures the glucose concentration in the interstitial fluid of the tissue every 5 minutes for up to 10.5 days and has a reportable range of 2.2 to 22.2 mmol/L. The corresponding receiver (Dexcom G7 receiver, Dexcom Inc) was synchronized with the local time and blinded so that the participants were not able to react to their glucose concentrations. Calibration was not required because the Dexcom G7 CGM system was calibrated by the manufacturer during production. After the study days, the sensor was removed and data were read out using the Dexcom Clarity software version 3.49.0 (Dexcom Inc).

### Glucose Metrics

This study examined 24 CGM-derived metrics based on consensus recommendations [23, 24]. Conventional glycemic parameters are mean, minimum and maximum glucose, the percentages of time spent in the euglycemic range (time in range [TIR], glucose 3.9-10 mmol/L), the hyperglycemic ranges (time above range [TAR10], glucose >10 mmol/L; TAR13.9, glucose >13.9 mmol/L), and the hypoglycemic ranges (time below range [TBR3.0], glucose <3.0 mmol/L; TBR3.9, glucose <3.9 mmol/L) and area under the curve (AUC).

#### Measures of glycemic variability

Conventional measures are SD and coefficient of variation (CV) [23, 24]. Additional measures are mean amplitude of glycemic excursion (MAGE), continuous overall net glycemic action (CONGA), and mean of daily differences (MODD), describing intraday and interday glycemic variability. They capture different aspects of glycemic variability.

- MAGE measures the intraday variation of glucose recordings. It is calculated as the mean of glucose excursions. A glucose excursion is included only if the difference between its maximum and minimum exceeded 1 SD from the arithmetic 24-hour mean. That means that MAGE considers especially higher excursions. Higher MAGE values indicate higher glycemic variability [25].
- CONGA also measures the intraday variation of glucose recordings. In contrast to MAGE, it does not ignore minor fluctuations in glucose values. Therefore, it is relevant for investigating glucose patterns in healthy individuals. It is the standard deviation of differences in measured glucose values, separated by n hours. It requires 24 hours or less of CGM. This study used a period of n = 24 hours. Higher CONGA values indicate higher glycemic variability [26].
- MODD measures the interday variation of glucose recordings. It is relevant because it allows assessing how consistent glucose patterns are from day to day. It is the mean of the difference between each glucose value and the value 24 hours before. Higher MODD values indicate higher variability [27].

#### Measures of glycemic control

A conventional measure is the glucose management indicator (GMI) as an estimate for the long-term glucose, based on the mean glucose concentration. Measures relevant for assessing severe hypoglycemia and severe hyperglycemia are the low blood glucose index (LBGI) and high blood glucose index (HBGI) [4, 23]. Additional measures (GRADE, COGI, M-value, J-Index) emphasize different aspects of glycemic control, as described later. These glucose metrics have also been used in studies with healthy populations, and normal ranges for young adults without diabetes are to be found in the study by Hill and colleagues [28].

- LBGI is a risk index for severe hypoglycemia. HBGI is a risk index for severe hyperglycemia. For LBGI, values less than 2.5 indicate a low risk of severe hypoglycemia, 2.5 to 5.0 a moderate risk, and values greater than 5.0 a high risk [29]. For the HBGI, values less than 4.5 indicate a low risk of severe hyperglycemia, 4.5 to 9 a moderate risk, and values greater than 9 a high risk [30].
- The glycemic risk assessment in diabetes equation (GRADE) is a risk score for assessing glycemic control [31]. A GRADE score less than 5 indicates a good glycemic control. The relevance of this measure is that it allows determining the relative contribution of hypoglycemia, euglycemia, and hyperglycemia to the GRADE score, calculated as “GRADE hypoglycemia,” “GRADE euglycemia,” and “GRADE hyperglycemia,” respectively [31].
- The continuous glucose monitoring index (COGI) is relevant for evaluating the quality of glucose control. It allows assessing the incremental benefit of different therapies. The index integrates 3 components of CGM data into 1 metric: hypoglycemia (TBR <3.9 mmol/L), euglycemia (TIR 3.9-10 mmol/L), and glycemic variability (SD), but not hyperglycemia. COGI therefore focuses more on the contribution of hypoglycemia than hyperglycemia to the glycemic control. COGI values range from 0 to 100, and higher values indicate a better glycemic control [32].
- The M-value was developed for measuring the ineffectiveness of an insulin treatment [33]. It is relevant for assessing greater glucose excursions, which are given greater weight. For calculating the M-value, one needs a reference glucose value, which can be freely chosen. This study used 5 mmol/L as reference value. A modified formula of the M-value was applied [34]. M-values between 0 and 18 indicate good control, between 19 and 31 adequate control, and above 32 poor control [33].
- The J-Index was developed from the M-value, but it is independent of a reference glucose value. It integrates mean glucose and SD. Since this index is weighted towards hyperglycemia, it is relevant for glucose profiles without episodes of severe or persistent hypoglycemia. A J-Index between 10 and 20 indicates an ideal control, between 20 and 30 a good control, between 30 and 40 a poor control, and greater than 40 a lack of control [35].

The CGM-derived metrics were calculated in this study for the whole 5-day period as well as separately for day and night blocks. Day and night blocks were categorized by using the individual bedtimes of the study participants. Since the calculation of CONGA and MODD requires a 24-hour period, it was not calculated separately for day and night blocks.

### Determination of Glycated Hemoglobin A_1c_

HbA_1c_ was determined to exclude volunteers with prediabetes or diabetes. Only participants with an HbA_1c_ less than 39 mmol/mol Hb were included in the study. HbA_1c_ was measured from capillary blood via turbidimetry at a wavelength of 700 nm using the HbA_1c_ test kit with the CUBE-S (Eurolyser Diagnostica GmbH). The LOT-depending measurement range is 20 to 130 mmol/mol Hb.

### Dietary Assessment

For assessing the individual dietary intake, all participants were instructed by an experienced nutritionist (A.K.) to complete a dietary record for 3 days (days 4-6). The participants recorded all food and drinks consumed and the times of consumption. They were asked to consume their usual food and drinks during this time period. They were encouraged to weigh their foods; however, to minimize the burden it was sufficient to use household units such as portion, teaspoon, or tablespoon. Each dietary record was analyzed using the nutritional PRODI 7 expert software, version 7.1 (Nutri-Science GmbH), calculating total energy intake (kJ/24 hours), amounts of macronutrients (carbohydrates, fat and protein), single glucose as well as total sugar that was defined as all monosaccharides and disaccharides. The energy and macronutrient intakes were further calculated as age-specific percentages of the recommended dietary allowance (RDA) from the German Nutrition Society for healthy people. In addition, the carbohydrate/fat ratio and the carbohydrate/protein ratio were calculated. All variables were separately determined for each of the 3 days of dietary recording and averaged over all 3 days.

### Statistical Analyses

Data were analyzed using SPSS for Windows version 29.0 (IBM) and R Studio version 4.2.3. Glucose metrics were determined for whole days and separately for day and night blocks using the R-package iglu [36]. Daytime CGM was defined by individual wake times (lights on until lights off) and nighttime CGM by individual sleeping times (lights off until lights on). Values are shown as means and standard deviations. Normal distribution of data was tested with the Shapiro-Wilk test. Differences between age groups were tested for statistical significance using *t* tests for independent samples or Mann-Whitney *U* tests. The relationship of glucose parameters with dietary parameters was analyzed by means of Pearson or Spearman rank correlation coefficients. Sex was not considered as a factor in the statistical analysis of data. Statistical significance was considered at *P* less than .050, and all statistical tests were performed 2-sided.

## Results

### General Characteristics and Nutrition of Study Participants

Table 1 shows the general characteristics and dietary intake of the young and older participants. The age groups did not differ with regard to BMI, but older adults had a statistically significantly larger abdomen circumference, higher body fat mass, and less body cell mass than young adults. The mean HbA_1c_ was significantly higher in the older adults. Furthermore, Table 1 presents for both age groups the intakes of energy and macronutrients as well as ratios between macronutrients averaged over 3 consecutive days. In both age groups, the mean energy intake met the RDA from the German Nutrition Society (young adults: 96.96 ± 17.25%, older adults: 95.17 ± 19.39%). Young and older adults consumed fewer carbohydrates (young adults: 66.33 ± 14.96%, older adults: 64.03 ± 16.46%), more fat (young: 118.66 ± 33.25%, older: 137.23 ± 43.39%), and more protein (young adults: 206.96 ± 103.55%, older adults: 120.48 ± 32.80%) than the RDA. The older adults consumed significantly less protein (−42.7 g/24 hours) and carbohydrates (−55.4 g/24 hours) than young adults did, but similar amounts of glucose and total sugar. Mean fat intake and energy intake did not significantly differ between age groups. The carbohydrate/fat ratio was significantly smaller in older adults than in young adults, while the carbohydrate/protein ratio did not differ between age groups.

**Table 1.** General characteristics and dietary intake of healthy study participants

Parameter	Young adults (n = 34)	Older adults (n = 27)	Group comparison
	Mean ± SD	Mean ± SD	*P^c^*
Age, y	26.3 ± 4.9	66.8 ± 5.0	**<.001**
BMI, kg/m^2^	23.5 ± 2.5	24.7 ± 2.6	.079
Abdomen circumference, cm	83.1 ± 6.7	93.2 ± 8.5	**<.001**
Body fat mass, %	23.7 ± 7.1	29.0 ± 5.7	**.003**
Body cell mass, kg	30.2 ± 6.8	24.6 ± 5.5	**.001**
HbA_1c_, mmol/mol Hb	33.5 ± 2.6	36.4 ± 1.7	**<.001**
Energy intake, kJ/24 h*^a^*	11 076 ± 2929	9324 ± 1879	.633
Carbohydrate intake, g/24 h*^a^*	275.6 ± 70.1	220.2 ± 53.0	**.001**
Total sugar intake, g/24 h*^a,b^*	109.3 ± 36.2	97.1 ± 24.2	.137
Glucose intake, g/24 h*^a^*	21.9 ± 11.3	18.0 ± 7.1	.212
Fat intake, g/24 h*^a^*	107.5 ± 35.8	107.1 ± 32.4	.988
Protein intake, g/24 h*^a^*	119.7 ± 63.4	77.0 ± 19.8	**.003**
Carbohydrate/Fat ratio*^a^*	2.92 ± 1.23	2.25 ± 0.71	**.008**
Carbohydrate/Protein ratio*^a^*	2.83 ± 1.20	3.05 ± 0.82	.410

Abbreviations: BMI, body mass index; Hb, hemoglobin; HbA_1c_, glycated hemoglobin A_1c_.

^a^Averaged over 3 consecutive days.

^b^Total sugar comprises all monosaccharides and disaccharides.

^c^ *T* tests for independent samples or Mann-Whitney *U* tests. Statistically significant age group differences are shown in bold.

### Age Group Comparisons of Glucose Metrics, Averaged Over 5 Whole Days

**Figure 1.**
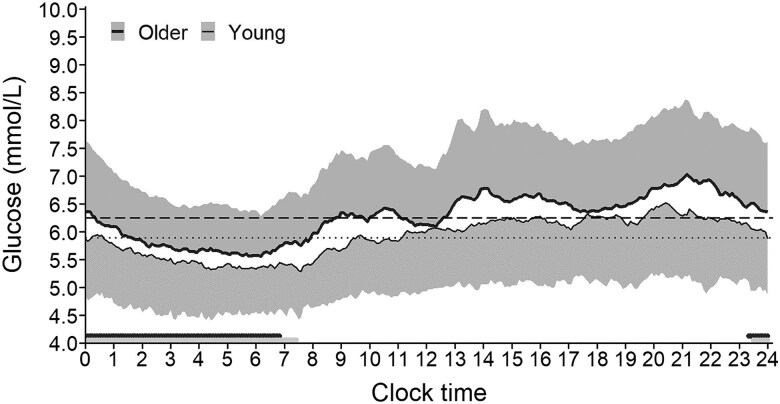
Mean glucose concentration with SD measured by continuous glucose monitoring (CGM) at 5-minute intervals in 34 healthy young adults (thin line, lower shaded area) and 27 healthy older adults (thick line, upper shaded area) with normal glycated hemoglobin A_1c_, averaged over five 24-hour days of CGM. The dotted (for young adults) and dashed (older adults) horizontal lines indicate the mean glucose value over 5 whole days. The horizontal bars above the x-axis represent mean sleeping times for young (gray bar) and older adults (black bar).

Fig. 1 presents the CGM profiles of young and older adults averaged over 5 consecutive whole days of recording. There were 85.29% CGM data for young adults and 96.30% CGM data for older adults. The older adults showed at each clock time higher mean glucose concentrations than young adults. For both age groups, daytime glucose concentrations were higher than nighttime concentrations. The variation of the glucose concentration, shown as SD in Fig. 1, was similar in the older and young adults throughout the whole day. During the hours of sleep, the glucose concentrations of both age groups fell steadily until reaching a minimum around 06:00 hours and then rose after awakening. Visual examination indicates for older adults from awakening until the evening 3 large glucose increases that occurred in the morning, at noon, and in the evening. The glucose rises increased from morning to evening. Thereafter, the glucose concentration decreased to the level of the 5-day mean glucose concentration or below. Young adults also showed 3 glucose increases, but these were less pronounced than in the older adults.

Table 2 displays glucose metrics by CGM for young and older adults averaged over the 5 whole days. Older adults had on average a significantly higher mean and minimum tissue glucose concentration than young adults, while maximum glucose concentration did not differ significantly between groups. The AUC was significantly higher in older compared with young adults. The glucose metrics for variability (SD, CV, MAGE, CONGA, and MODD) and TIR did not significantly differ between age groups. Metrics related to hyperglycemia (HBGI and GRADE hyperglycemia) were significantly higher in older adults, and those related to hypoglycemia (TBR, LBGI, and GRADE hypoglycemia) significantly higher in young adults. Both age groups had a low risk of severe hyperglycemia (HBGI < 4.5) and a low risk of severe hypoglycemia (LBGI < 2.5). In both age groups, glycemic control was good (GRADE < 5, M-value ≤18) or ideal (J-Index ≤20). Nevertheless, most indicators of glycemic control (GMI, GRADE, M-value, and J-Index) were significantly higher in older compared with young adults, indicating a somewhat poorer glycemic control in older adults.

**Table 2.** Glucose metrics in healthy young and older adults, averaged over 5 whole days of continuous glucose monitoring

Glucose parameter*^a^*	Young adults (n = 34)	Older adults (n = 27)	Group comparison
	Mean ± SD	Mean ± SD	*P^b^*
**Conventional parameters**			
Mean glucose, mmol/L	5.89 ± 0.55	6.25 ± 0.65	**.023**
Minimum glucose, mmol/L	3.19 ± 0.80	3.70 ± 0.90	**.034**
Maximum glucose, mmol/L	10.18 ± 1.23	10.42 ± 1.47	.850
TAR 13.9 mmol/L, %	0.00 ± 0.00	0.01 ± 0.07	—
TAR 10 mmol/L, %	0.28 ± 0.35	0.75 ± 1.37	.744
TIR 3.9-10 mmol/L, %	97.55 ± 4.01	98.37 ± 2.03	.621
TBR 3.9 mmol/L, %	2.17 ± 4.05	0.88 ± 1.79	.082
TBR 3.0 mmol/L, %	0.32 ± 0.73	0.10 ± 0.27	**.026**
AUC, mmol × h/L	5.89 ± 0.55	6.25 ± 0.65	**.022**
**Parameters for glycemic variability**			
SD, mmol/L	0.93 ± 0.22	0.99 ± 0.21	.353
CV, %	15.86 ± 4.04	15.87 ± 3.22	.581
MAGE, mmol/L	2.36 ± 0.55	2.61 ± 0.56	.172
CONGA, mmol/L	1.16 ± 0.28	1.13 ± 0.25	.561
MODD, mmol/L	0.87 ± 0.23	0.83 ± 0.18	.486
**Parameters for glycemic control**			
GMI, %	5.85 ± 0.24	6.01 ± 0.28	**.023**
LBGI	1.09 ± 0.96	0.67 ± 0.76	**.034**
HBGI	0.30 ± 0.25	0.55 ± 0.48	**.015**
GRADE	1.66 ± 0.83	2.15 ± 1.06	**.046**
GRADE hypoglycemia, %	11.49 ± 16.69	6.32 ± 12.84	**.017**
GRADE euglycemia, %	72.44 ± 16.33	66.29 ± 13.70	.122
GRADE hyperglycemia, %	18.83 ± 11.50	29.25 ± 15.08	**.003**
COGI	93.66 ± 11.20	96.90 ± 5.08	.256
M-value	1.74 ± 1.14	2.66 ± 1.87	**.031**
J-Index	15.23 ± 2.72	17.19 ± 3.49	**.016**

Abbreviations: AUC, area under curve; CGM, continuous glucose monitoring; COGI, continuous glucose monitoring index; CONGA, continuous overall net glycemic action; CV, coefficient of variation; GMI, glucose management indicator; GRADE, glycemic risk assessment in diabetes equation; HBGI, high blood glucose index; LBGI, low blood glucose index; MAGE, mean amplitude of glycemic excursion; MODD, mean of daily differences; TAR, time above range; TBR, time below range; TIR, time in range.

^a^Averaged over 5 whole days of CGM, based on individual waketimes and sleep times.

^b^ *T* tests for independent samples or Mann-Whitney *U* tests. Statistically significant age group differences are shown in bold.

### Age Group Comparisons of Glucose Metrics, Separately for Day and Night Blocks

**Figure 2.**
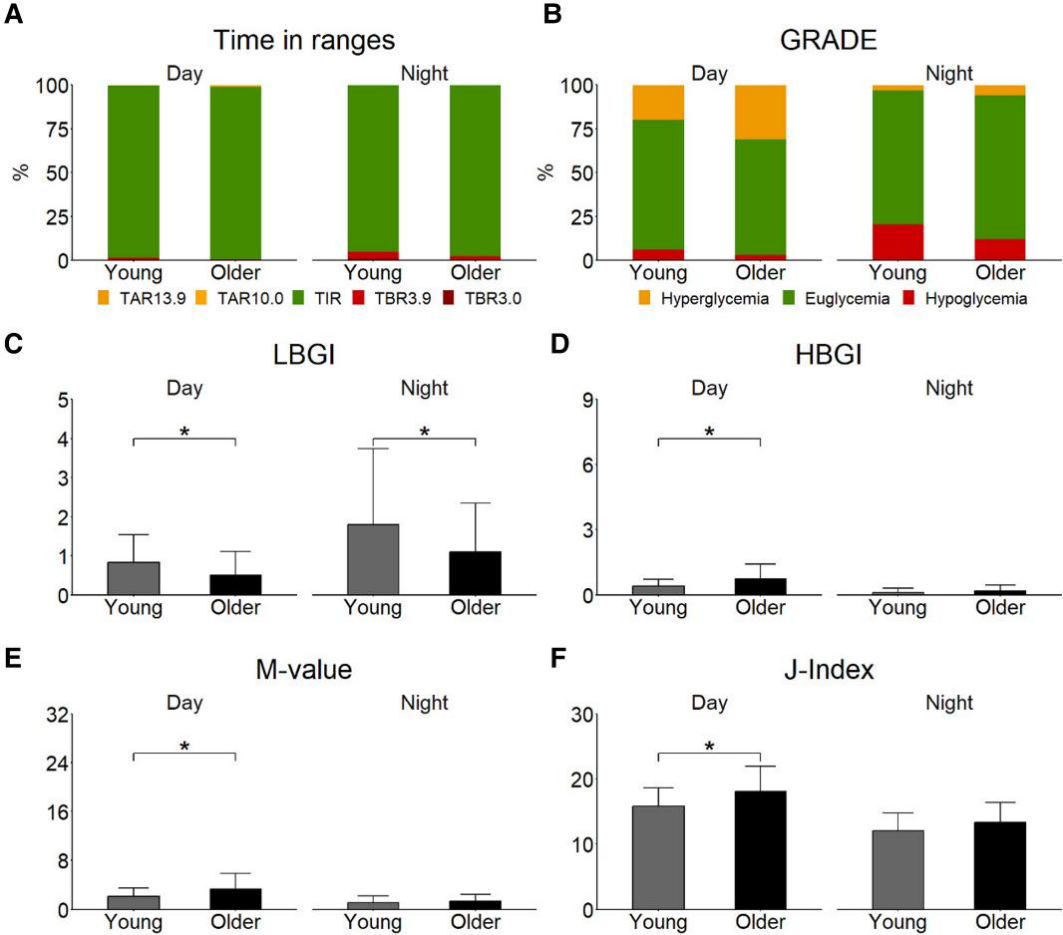
Comparison of 34 young and 27 older healthy participants with respect to day and night blocks of continuous glucose monitoring averaged over 5 consecutive days. Metrics for time in euglycemic/hypoglycemic/hyperglycemic ranges and metrics for glycemic control (GRADE, LBGI, HBGI, M-value, and J-Index) are presented. Means and SDs are shown for A, percentages of time in ranges (TAR, time in hyperglycemic range; TIR, time in euglycemic range, TBR, time in hypoglycemic range); B, contribution of hyperglycemia, euglycemia, and hypoglycemia to the GRADE score; C, LBGI (risk score for severe hypoglycemia); D, HBGI (risk score for severe hyperglycemia); E, M-value; and F, J-Index. Lower values in the y-axis of panels C to F indicate better glycemic control. The scaling of the y-axis indicates categories of low to moderate risk of C, severe hypoglycemia or D, severe hyperglycemia, E, categories of adequate to good glycemic control or F, categories of good to ideal glycemic control. Asterisks indicate statistically significant differences between age groups. *P less than .050.

The glucose metrics were analyzed not only for whole days, but also separately for day and night blocks of the 5 days of CGM recording. Fig. 2 displays the glucose metrics for day and night blocks, separately for young and older adults. The diurnal and nocturnal glucose concentrations of both age groups were predominantly in the euglycemic target range of 3.9 to 10 mmol/L (TIR), to a lower extent in the hypoglycemic range below 3.9 mmol/L (TBR), and to a minimal extent in the hyperglycemic range above 10 mmol/L (TAR) (Fig. 2A). The glycemic variability was lower at night than during the day in both age groups, as indicated by a lower SD, a lower CV, and a lower MAGE at night (each *P* < .001). The risk of hypoglycemia was higher at night than during the day in both age groups, as shown by a higher nocturnal proportion of hypoglycemia of the GRADE score (*P* < .001) and a higher LBGI (*P* < .001) (Fig. 2C). The risk of hyperglycemia was lower at night than during the day in both age groups, as shown by a lower nocturnal proportion of hyperglycemia of the GRADE score (*P* < .001) and a lower HBGI (*P* < .001) (Fig. 2D). The glycemic control was better at night than during the day in both age groups, as indicted by a higher nocturnal proportion of euglycemia of the GRADE score (young adults: *P* = .002; older adults: *P* < .001; Fig. 2B) and a lower nocturnal M-value and a lower nocturnal J-Index (*P* < .001). Nevertheless, both age groups had at night and during the day good glycemic control (M-value ≤18; Fig. 2E) or ideal glycemic control (J-Index ≤20; Fig. 2F).

A comparison of the nocturnal glucose metrics shows no differences between age groups with respect to mean glucose concentration, glycemic variability, and glycemic control (Supplementary Table S1 [37]). The only nocturnal difference was a significantly higher minimum glucose concentration in older compared with young adults. A comparison of the daytime glucose parameters also shows no difference in glycemic variability between age groups, but revealed a significantly higher mean glucose concentration and a significantly poorer glycemic control in older adults compared with young adults. The poorer glycemic control of the older group is indicated by a higher GMI, a lower proportion of “GRADE euglycemia,” a higher risk of hyperglycemia (higher HBGI and higher GRADE score), a higher M-value, and a higher J-Index.

### Relationships of Glucose Metrics With Dietary Parameters by Age Groups, Averaged Over 3 Days

Because the CGM metrics for hypoglycemic ranges (TBR3.0 and TBR3.9) and the hyperglycemic ranges (TAR10 and TAR13.9), for “GRADE hypoglycemia” and “GRADE hyperglycemia” varied only slightly, no correlation analyses were performed.

**Figure 3.**
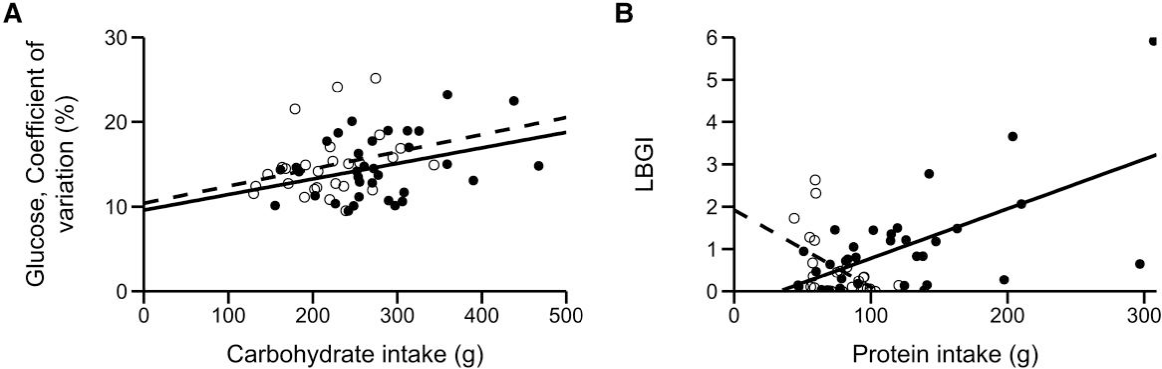
Relationship of A, carbohydrate intake with glucose variability (coefficient of variation) and of B, protein intake with the hyperglycemic risk (LBGI) in 34 young (black circles) and 27 older (white circles) healthy adults. The carbohydrate intake correlated in both age groups positively with glycemic variability (young adults: *P* = .043; older adults: *P* = .047). The protein intake correlated positively with the hypoglycemic risk in young adults (*P* = .002) and negatively in older adults (*P* = .009). Regression lines for young adults (solid lines) and older adults (dashed lines) are shown.

The carbohydrate intake correlated positively with glycemic variability both in young adults (CV; *P* = .043) and older adults (CV; *P* = .047; SD, *P* = .034) (Supplementary Table S2 [37]; Fig. 3A). Only in young adults, a higher carbohydrate intake was associated with poorer glycemic control (COGI; *P* = .006; GRADE euglycemia; *P* = .014) and thus a shorter time in the euglycemic range (TIR; *P* = .004). No age group displayed any statistically significant correlation between the intake of total sugar, glucose or fat and CGM metrics.

The carbohydrate/fat ratio correlated positively with glycemic variability (MAGE; *P* = .023) in older adults (Supplementary Table S2 [37]). A higher carbohydrate/fat ratio was associated with poorer glycemic control (GRADE euglycemia; *P* = .006) in young adults.

Protein intake did not correlate with any parameter of glycemic variability in any age group, but it was associated with CGM metrics for glycemic control (Supplementary Table S2 [37]). Here, a higher protein intake was related with a higher risk of hypoglycemia in young adults (LBGI; *P* = .002), but with a lower risk of hypoglycemia in older adults (LBGI, *P* = .009) (Fig. 3B). Additional CGM parameters for glycemic control showed inconsistent relationships with protein intake in young adults. Here, a higher protein intake was associated with poorer glycemic control (COGI; *P* = .018) or with better glycemic control (GMI; *P* = .004; J-Index; *P* = .017).

The carbohydrate/protein ratio correlated positively with glycemic variability (SD, *P* = .042; CV; *P* = .006; MAGE; *P* = .023) in older adults (Supplementary Table S2 [37]). In young adults, a higher carbohydrate/protein ratio was associated with poorer glycemic control (GMI; *P* = .001; GRADE; *P* = .039; M-value; *P* = .046; J-Index; *P* = .003), in particular with a higher risk of hyperglycemia (HBGI; *P* = .026), but also with a lower risk of hypoglycemia (LBGI; *P* = .001).

## Discussion

### Applicability of Different Glucose Metrics

This study provided CGM-derived metrics for determining glucose variability and glycemic control in healthy young and older adults. As described in “Materials and Methods,” the different glucose metrics capture different aspects of glycemic variability and control. It is important to note that these parameters are not interchangeable. It is suggested to consider this in future studies. There are guidelines for patients presenting with diabetes that recommend using the glucose metrics SD, CV, GMI, and LBGI/HBGI [23, 24]. The use of these glucose metrics in healthy individuals will facilitate comparisons between patients with diabetes and healthy individuals. Nevertheless, the other glucose metrics are also of interest because they offer the possibility to investigate additional aspects of glycemic variability and glycemic control.

### Age Group Comparisons in Glucose Metrics

This study provides the first comparison of CGM-derived glucose metrics between healthy young and older adults for whole days and day and night blocks. The 5-day CGM-derived data for mean glucose, glycemic variability, and glycemic control of both age groups are within the recommended ranges for adults without diabetes [28, 38]. The CGM metrics indicate for both healthy age groups good to ideal glycemic control and low risks for severe hypoglycemia and hyperglycemia.

The mean glucose concentration of the healthy older adults is slightly higher than that reported in previous studies for this age group [16, 38]. This can be explained by the individual quantities of food consumed in the present study, compared with standardized meals in earlier studies. Therefore, the present findings more clearly reflect glucose profiles from real living conditions of healthy older people. A comparison between older and young healthy adults shows a higher mean glucose concentration in older adults, in line with the known decline in insulin sensitivity during healthy aging [39].

The glycemic variability observed in the healthy older adults is comparable to that reported in apparently healthy older individuals [13, 16, 40]. Interestingly, the present findings show that glycemic variability does not differ for any of the 5 CGM-derived measures between the young and older adults, neither intraday nor interday variability. This suggests that healthy aging may not affect glycemic variability. Previous CGM studies reported inconsistent results on the influence of age on glycemic variability, possibly because they investigated groups of mixed ages [41, 42].

Furthermore, this study found that the healthy older adults, compared with young adults, spent more time in the hyperglycemic range and less time in the hypoglycemic range, confirming most previous research [15, 38]. In addition to glycemic ranges, the present study is the first to provide several CGM-derived metrics of glycemic control from healthy adults older than 60 years. Four of 5 compound metrics for overall glycemic control indicate a significantly poorer glycemic control in older compared with young adults. However, metrics that assess separately the risks for hyperglycemia and hypoglycemia consistently indicate a higher risk of hyperglycemia and a lower risk of hypoglycemia in older compared with young adults. This emphasizes the need for using not only compound parameters for glycemic control, but also using separate measures for analyzing the specific risks for hypoglycemia and hyperglycemia. This would also be consistent with the definition of glycemic control to maintain glucose concentrations in the euglycemic range and to avoid both hypoglycemic and hyperglycemic episodes [2]. Taken together, the present findings indicate that healthy aging is primarily affected by changes in glycemic control, but not in glycemic variability. This implies that future studies on healthy aging should focus on changes in glycemic control including the risks for hypoglycemia and hyperglycemia.

### Day-Night Comparisons in Glucose Metrics

An international consensus group on CGM metrics recommended analyzing glucose metrics by day and night blocks [24]. This requires defining day and night blocks. The present study used the individual sleep-wake times of the study participants while previous studies used fixed clock times [40, 43]. We recommend future studies use individual sleep-wake times when comparing data from young and older adults because of the earlier sleep-wake timing in older relative to young adults. Reference values for CGM-derived glucose metrics for day and night blocks are currently missing from healthy adults. The present study is the first to provide such data that may serve as healthy control data for comparison with data from patients presenting with obesity, prediabetes, or diabetes.

The comparison of day and night blocks revealed that both age groups had lower glycemic variability, lower risk of hyperglycemia, higher risk of hypoglycemia, and better glycemic control during the sleeping period, compared with the waking period. This can be explained by the nocturnal fasting. A comparison between age groups showed that most differences in glucose metrics between older and young adults were present during the waking period where older adults had a higher mean glucose concentration and a poorer glycemic control. This can be explained by the decline in glucose tolerance with aging [19]. Although nocturnal mean glucose concentration did not differ significantly between age groups, the older adults had a slightly higher mean glucose concentration than young adults throughout the whole night (cf, Fig. 1) that agrees with previous results [40, 43]. A further finding was that older adults, compared with young adults, had a higher nocturnal minimum glucose concentration. This may reduce their nocturnal risk of hypoglycemia and could be explained by the reduction in insulin secretion and sensitivity during aging [39].

### Relationship of Glucose Metrics With Diet

Studies on the effect of dietary intake of carbohydrates, proteins, and fats on glycemic variability and glycemic control by CGM are missing for healthy adults older than 60 years. This study found both for older and young adults a positive relationship between carbohydrate intake and glycemic variability (cf, Fig. 3A). This agrees with results in normoglycemic individuals of mixed ages [21]. The present study found no relationship between protein intake and glycemic variability. This contradicts a previous result of a negative relationship [21]. A possible reason for the differing results is that the authors used CGM data from whole days, contrary to CGM data from daytime in the present study. In addition, differing meal patterns may contribute to the observed differences [44]. The effect of proteins on glycemic variability may therefore be complex and should be investigated in detail in future studies.

The influence of diet on CGM-derived metrics for glycemic control has not yet been studied in healthy older adults. This study found that a higher dietary intake of protein was related to a lower risk of hypoglycemia in older adults, but to a higher risk in young adults (cf, Fig. 3B). In addition, a higher carbohydrate/protein ratio was associated with a lower risk of hypoglycemia in young adults. An explanation for the negative relationship of protein with glucose concentration in young adults is that dietary protein increases glucose disappearance [45]. The observed age group differences could be explained by the age-related decrease of the glucose disposal rate and the decrease in digestion and absorption of proteins [46]. In addition, the present older adults consumed significantly smaller amounts of protein than young adults did. Moreover, fewer older adults followed a vegetarian (11.1%) or vegan (0%) diet, compared with young adults (vegetarian: 35.3%; vegan: 5.9%). This may be important because studies have shown that the type of protein (animal vs plant based) affects the risk of developing type 2 diabetes [47]. However, the study sample was too small to examine this effect on glycemic control. Future studies are warranted to explore the effect of vegetarian and vegan diet on glucose metrics by CGM in different age groups.

Regarding fat intake, the carbohydrate/fat ratio was significantly smaller in older adults due to a lower intake of carbohydrates. While carbohydrate intake correlated positively and significantly with glucose variability (SD and CV) in older adults, this is no longer the case when using the carbohydrate/fat ratio. An explanation is that fat retards gastric emptying, thereby slowing down the glucose uptake from the intestine into the blood, resulting in a weaker increase in blood glucose concentration. This emphasizes the relevance of using ratios to obtain information on the combined effect of macronutrients on glucose metrics. Notably, this effect is not observed with MAGE, suggesting that the influence of fat intake may be more pronounced in smaller glucose excursions.

### Strengths and Limitations of the Study

The present study has some strengths. One strength is that all study participants were free of diseases and medications, and did not present with prediabetes or obesity. A second strength is that the individual sleeping times were used to define daytime and nighttime blocks. This allows more precise analysis of changes in glucose metrics between sleeping and waking periods. Previous studies used fixed time blocks that did not consider the individual sleep-wake times. A third strength is the use and comparison of different aspects of glycemic variability and glycemic control. This study also has some limitations. One limitation is that insulin was not measured to investigate differences in insulin metabolism and possible influences on glucose parameters. The measurement of insulin was not possible because venous blood sampling was unavailable in the healthy volunteers due to invasiveness. A second limitation is that the physical activity level of the participants could not be determined during the study days, although it may influence the glucose metrics.

### Conclusions

This study found that CGM-derived indicators of intraday and interday glycemic variability did not differ between healthy older and young adults. This suggests that healthy aging will not increase glycemic variability. Glycemic control was good in both age groups, but statistically significantly better in young adults. The risk of hyperglycemia increases and the risk of hypoglycemia decreases during healthy aging. Age group differences are present during the day, but disappear during the night, except for a lower nocturnal risk of hypoglycemia in older adults. The dietary intake of carbohydrates was related to glycemic variability and that of protein to glycemic control. In both age groups, higher carbohydrate intake was significantly associated with higher glycemic variability. Higher protein intake was related to lower hypoglycemic risk in older adults, but to higher hypoglycemic risk in young adults. This suggests that the effect of dietary protein on hypoglycemia may change during healthy aging. Looking ahead, standardized glucose metrics by CGM for healthy adults should be reported in future studies to ensure better comparability. Here, the use of different CGM metrics for glycemic variability and glycemic control offers the possibility of gaining more insight into different aspects of glucose metabolism that may change during healthy aging.

## Data Availability

Some or all data sets generated during and/or analyzed during the current study are not publicly available but are available from the corresponding author on reasonable request.

## Abbreviations

AUC: area under curve
CGM: continuous glucose monitoring
COGI: continuous glucose monitoring index
CONGA: continuous overall net glycemic action
CV: coefficient of variation
GMI: glucose management indicator
GRADE: glycemic risk assessment in diabetes equation
HbA_1c_: glycated hemoglobin A_1c_
HBGI: high blood glucose index
LBGI: low blood glucose index
MAGE: mean amplitude of glycemic excursion
MODD: mean of daily differences
RDA: recommended dietary allowance
TAR: time above range
TBR: time below range
TIR: time in range
